# Childhood trauma is associated with developmental trajectories of EEG coherence, alcohol-related outcomes, and PTSD symptoms

**DOI:** 10.1017/S0033291724002599

**Published:** 2024-11

**Authors:** Zoe E. Neale, Kaitlin Bountress, Christina Sheerin, Stacey Saenz de Viteri, Shannon Cusack, David Chorlian, Peter B. Barr, Isabelle Kaplan, Gayathri Pandey, Kristina A. Osipenko, Vivia McCutcheon, Sally I-Chun Kuo, Megan E. Cooke, Sarah J. Brislin, Jessica E. Salvatore, Chella Kamarajan, COGA Investigators, B. Porjesz, V. Hesselbrock, T. Foroud, A. Agrawal, D. Dick, V. Hesselbrock, H.J. Edenberg, T. Foroud, Y. Liu, M.H. Plawecki, S. Kuperman, J. Kramer, J. Meyers, C. Kamarajan, A. Pandey, L. Bierut, J. Rice, K. Bucholz, A. Agrawal, M. Schuckit, J. Tischfield, R. Hart, J. Salvatore, L. Almasy, A. Goate, P. Slesinger, D. Scott, L. Bauer, J. Nurnberger, L. Wetherill, X. Xuei, D. Lai, S. O'Connor, G. Chan, D.B. Chorlian, J. Zhang, P. Barr, S. Kinreich, Z. Neale, G. Pandey, N. Mullins, A. Anokhin, S. Hartz, E. Johnson, V. McCutcheon, S. Saccone, J. Moore, F. Aliev, Z. Pang, S. Kuo, A. Merikangas, H. Chin, A. Parsian, Ting-Kai Li, P. Michael Conneally, Raymond Crowe, Wendy Reich, Bernice Porjesz, Ananda B. Amstadter, Jacquelyn L. Meyers

**Affiliations:** 1 Department of Psychiatry and Behavioral Sciences, State University of New York Downstate Health Sciences University, Brooklyn, NY, USA; 2 VA New York Harbor Healthcare System, Brooklyn, NY, USA; 3 Department of Psychiatry, Virginia Commonwealth University, Virginia Institute for Psychiatric and Behavior Genetics, Richmond, VA, USA; 4 Department of Psychiatry, Washington University School of Medicine, St Louis, MO, USA; 5 Department of Psychiatry, Rutgers University, Robert Wood Johnson Medical School, Piscataway, NJ, USA; 6 The Collaborative Study on the Genetics of Alcoholism (COGA) funded by the NIAAA (U10AA008401)

**Keywords:** alcohol use disorder, childhood trauma, EEG coherence, neurodevelopment, posttraumatic stress disorder

## Abstract

**Background:**

Associations between childhood trauma, neurodevelopment, alcohol use disorder (AUD), and posttraumatic stress disorder (PTSD) are understudied during adolescence.

**Methods:**

Using 1652 participants (51.75% female, baseline *M*_age_ = 14.3) from the Collaborative Study of the Genetics of Alcoholism, we employed latent growth curve models to (1) examine associations of childhood physical, sexual, and non-assaultive trauma (CPAT, CSAT, and CNAT) with repeated measures of alpha band EEG coherence (EEGc), and (2) assess whether EEGc trajectories were associated with AUD and PTSD symptoms. Sex-specific models accommodated sex differences in trauma exposure, AUD prevalence, and neural development.

**Results:**

In females, CSAT was associated with higher mean levels of EEGc in left frontocentral (LFC, ß = 0.13, *p* = 0.01) and interhemispheric prefrontal (PFI, ß = 0.16, *p* < 0.01) regions, but diminished growth in LFC (ß = −0.07, *p* = 0.02) and PFI (ß = −0.07, *p* = 0.02). In males, CPAT was associated with lower mean levels (ß = −0.17, *p* = 0.01) and increased growth (ß = 0.11, *p* = 0.01) of LFC EEGc. Slope of LFC EEGc was inversely associated with AUD symptoms in females (ß = −1.81, *p* = 0.01). Intercept of right frontocentral and PFI EEGc were associated with AUD symptoms in males, but in opposite directions. Significant associations between EEGc and PTSD symptoms were also observed in trauma-exposed individuals.

**Conclusions:**

Childhood assaultive trauma is associated with changes in frontal alpha EEGc and subsequent AUD and PTSD symptoms, though patterns differ by sex and trauma type. EEGc findings may inform emerging treatments for PTSD and AUD.

## Introduction

Childhood trauma is common, with an estimated ~20–66% of individuals in the United States experiencing at least one traumatic event before adulthood (Blaustein, 2013; Finkelhor, Turner, Ormrod, & Hamby, 2009; Read, Ouimette, White, Colder, & Farrow, 2011). Childhood trauma encompasses interpersonal victimization (e.g. physical, sexual abuse/violence) as well as non-interpersonal events (e.g. accidents, illness, loss; Briggs-Gowan, Carter, & Ford, 2012; Mongillo, Briggs-Gowan, Ford, & Carter, 2009). Exposure to childhood trauma is thought to disrupt ‘normative’ stages of childhood development, including cognitive, emotional, and social skills development, and predisposes children to psychiatric sequelae (D'Andrea, Ford, Stolbach, Spinazzola, & van der Kolk, 2012; Mongillo et al., 2009; Teicher & Samson, 2013), including posttraumatic stress disorder (PTSD) (Duncan, Saunders, Kilpatrick, Hanson, & Resnick, 1996; Khoury, Tang, Bradley, Cubells, & Ressler, 2010) and alcohol use disorder (AUD) (Schückher, Sellin, Fahlke, & Engström, 2018). Experiencing childhood trauma yields PTSD and AUD hazard ratios of ~1.4–3.5 (Sartor et al., 2011, 2012) thus, it is important to improve our understanding of the mechanisms by which exposure to trauma may impact development and psychiatric outcomes.

Epidemiologic studies report high co-occurrence between PTSD and both problematic alcohol use and AUD (Brown, Stout, & Mueller, 1999; Debell et al., 2014; Kessler, Chiu, Demler, Walters, & Walters, 2005; Mills, Teesson, Ross, & Peters, 2006; Pietrzak, Goldstein, Southwick, & Grant, 2011). One model for understanding this comorbidity is the shared liability model, which suggests that shared, common factors (e.g. trauma, genetics, physiological, and psychological traits) contribute to increased risk for alcohol use behaviors, AUD, and PTSD (Danovitch, 2016). Indeed, twin and molecular studies suggest correlated genetic risk (Bountress et al., 2022; Sartor et al., 2011; Sheerin et al., 2020; Xian et al., 2000). Growing research has begun to explore the role of shared neurobiological mechanisms (Brady & Sinha, 2005; Gilpin & Weiner, 2017). Neurophysiological and neuropsychological measurement can further our understanding of shared risk beyond self-report data (Davis et al., 2013). Some work has focused on the role of early stress in producing a cascade of neurobiological changes, such as reduced functional activity and/or structural alterations in key brain areas, that may act to increase risk for psychiatric disorders (Teicher et al., 2003). More research is needed to uncover possible mechanisms by which early trauma may influence neuropsychological development.

Electroencephalogram (EEG) has long been used to examine individual differences in brain function and neuropsychiatric health (Smit et al., 2021). EEG coherence (EEGc) is a heritable measure of neural functional connectivity, which measures the degree of synchrony in brain oscillatory activity between two regions (Chorlian, Rangaswamy, & Porjesz, 2009; Chorlian et al., 2007; Markovska-Simoska, Pop-Jordanova, & Pop-Jordanov, 2018). The human central nervous system has a prolonged developmental course, with critical periods in early life and adolescence, especially in prefrontal regions (Larsen & Luna, 2018; Silbereis, Pochareddy, Zhu, Li, & Sestan, 2016). Childhood stress can harm neural development during these sensitive periods (Andersen et al., 2008). Increased neural functional connectivity, measured via EEG, has been observed in cross-sectional studies of childhood trauma (Cook, Ciorciari, Varker, & Devilly, 2009), and adults with AUD and PTSD (Almli et al., 2018; Dunkley et al., 2015; Huang, Mohan, De Ridder, Sunaert, & Vanneste, 2018; Park et al., 2017), suggesting that increased functional connectivity may be a shared pathway of risk for AUD and PTSD. Associations between EEGc and neuropsychiatric conditions are complex, but better characterized in schizophrenia (Maran, Grent-; -Jong, & Uhlhaas, 2016), where greater connectivity in lower frequency bands (delta and theta) corresponds to lower cognitive performance and abnormal cortical organization (Di Lorenzo et al., 2015; Lehmann et al., 2014). However, increased coherence is not always adverse – frontal alpha connectivity, involved in information processing and attention (Foxe & Snyder, 2011), is lower in schizophrenia compared to controls (e.g. Di Lorenzo et al., 2015; Lehmann *et al*. 2014; Tauscher, Fischer, Neumeister, Rappelsberger, & Kasper, 1998). Prior research from the Collaborative Study on the Genetics of Alcoholism (COGA) indicates that AUD manifests as increased resting EEG interhemispheric theta and alpha coherence in fronto-central, fronto-temporal, temporo-parietal, centroparietal and parietal-occipital regions (Meyers et al., 2021; Porjesz & Rangaswamy, 2007; Rangaswamy & Porjesz, 2008). Another COGA study indicated that AD polygenic scores were associated with increased fronto-central, temporo-parietal, centroparietal, and parietal-occipital interhemispheric theta and alpha connectivity in males (Meyers et al., 2019a). This research on EEGc could be useful in investigating a possible shared pathway of risk for AUD and PTSD.

Research on the relationship between trauma and neural functional connectivity has focused almost exclusively on adults, ignoring periods of rapid EEGc development in adolescence and young adulthood (Cook et al., 2009; Thatcher, North, & Biver, 2008). Normative developmental trajectories of EEGc in adolescence have not been comprehensively characterized to our knowledge (Segalowitz, Santesso, & Jetha, 2010) as there are only a few studies with smaller samples; however, emerging literature from COGA suggests alpha coherence tends to increase throughout adolescence, flatten around mid-twenties, and then slowly decline (Chorlian et al., 2024). Disrupted alpha rhythm, power, and frontal asymmetry have also been detected in individuals with a range of psychiatric conditions (Eidelman-Rothman, Levy, & Feldman, 2016; Ippolito et al., 2022; Périard et al., 2024), including PTSD in adults (Badura-Brack et al., 2015; Huang et al., 2014; Kemp et al., 2010; Meyer et al., 2015; Popescu et al., 2019). Alpha frequency EEGc is of particular interest as alpha activity has been implicated in studies of impaired memory and cognitive decline in older adults (Babiloni et al., 2018; Blinowska et al., 2017; Hogan, Swanwick, Kaiser, Rowan, & Lawlor, 2003; Zhang et al., 2021), and is hypothesized to be related to acetylcholine levels in the brain (Babiloni et al., 2013; Sharma & Nadkarni, 2020). Childhood trauma is associated with increased right alpha EEG asymmetry (Meiers, Nooner, De Bellis, Debnath, & Tang, 2020), which has also been linked to low mood and social withdrawal in adolescence (Stewart, Towers, Coan, & Allen, 2011). Yet, only a single, small study of EEG functional connectivity and trauma in adolescence has been published (Cook et al., 2009). Correlations of brain connectivity measures with attention and psychopathology suggest that increased EEG functional connectivity contributes to deficits in cognitive functioning and psychopathology (Canuet et al., 2011; Imperatori et al., 2015; Kamarajan et al., 2020; Zinn, Zinn, & Jason, 2016), but without longitudinal data, cannot be definitively tested. To the best of our knowledge, EEGc specifically has not yet been examined longitudinally as a biological marker that may link trauma exposure to alcohol-related outcomes and PTSD in adolescents.

The purpose of this study is to investigate the relations among childhood trauma, trajectories of EEG functional connectivity, and risk for AUD and PTSD in adolescence and young adulthood. Using data from COGA, a multi-site study of extended families densely affected with alcohol-related problems (Agrawal et al., 2023; Meyers et al., 2023), we examined associations between childhood trauma, longitudinal EEGc trajectories, and downstream associations between EEGc and alcohol and trauma-related outcomes (i.e. PTSD symptoms) in COGA's prospective study of adolescent and young adult offspring. We hypothesized that childhood trauma would be associated with differences in frontal alpha EEGc, with the most robust effects observed for those individuals with history of childhood sexual assault. We also expected that these differences in functional connectivity would be associated with increased AUD and PTSD symptoms. We hope findings increase understanding of long-term effects of childhood trauma on neural functioning and psychiatric and behavioral outcomes, enhance knowledge of biological mechanisms by which trauma influences outcomes, and advance work towards novel opportunities for intervention targets.

## Methods

### Sample

**Figure 1.**
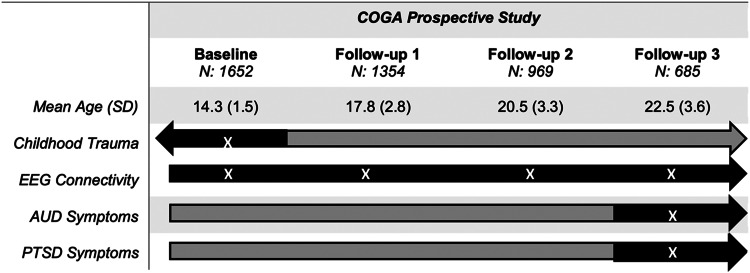
This flowchart illustrates the COGA assessments used in the present study. Gray bars represent data collected in COGA, and black bars and white ‘X’ represent assessment waves used in the current study. The analytic sample was limited to individuals aged 12–16 at baseline.

The Collaborative Study on the Genetics of Alcoholism (COGA)'s prospective study began its multi-site data collection in 2004 and ended in 2019. Details on data collection and procedures have been published previously (Dick et al., 2023). Briefly, 3715 offspring (14 495 total assessments) from families densely affected with AUD and community comparison families who had at least one parent interviewed in an earlier phase of the COGA study, were enrolled when they were between the ages of 12–22, with new participants added as they reached the age of 12. The COGA study re-assessed participants approximately every two years. A comprehensive battery was administered that included the adult Semi-Structured Assessment for the Genetics of Alcoholism (SSAGA) or the age-appropriate adolescent SSAGA (cSSAGA-A) and parent version (cSSAGA-P) if participants were under age 18. The SSAGA and cSSAGA-A covered substance use problems as well as other psychiatric disorders, including PTSD. As previously detailed (Meyers et al., 2023), a brain function battery was administered at each assessment. These included neurophysiological measures during resting state. Figure 1 shows available data and which assessments are used in the present study. Due to differences in EEG functional connectivity during adolescence compared to adulthood (Segalowitz et al., 2010), and to account for the mean age of onset of regular drinking for the sample (17.48 for males and 17.85 for females), the analytic sample was limited to adolescents age 12–16 at baseline with three or more EEG assessments (*n* = 1652).

### Assessment of primary constructs

Lifetime history of 21 potentially traumatic events (DSM-IV Criterion A) were based on cSSAGA-A baseline assessments. Although the SSAGA or cSSAGA-A was given at all assessment timepoints, participants were asked to report *lifetime maximum* DSM-5 AUD symptom count (AUDsx; range 0–11) and, for those who endorsed a Criterion A event, *lifetime maximum* DSM-IV PTSD Criterion B-D symptom count scores (PTSDsx; range 0–20) at each wave. Therefore, in this study we used lifetime symptom counts ascertained at follow-up 3 (*M*_age_ = 22) to allow sufficient opportunity for AUDsx to present in late adolescence/young adulthood. Based on evidence that interpersonal assaultive events are more ‘potent’ than non-assaultive events, that traumatic events cluster together, and to remain consistent with prior studies (Meyers et al., 2019b; Subbie-Saenz de Viteri et al., 2020), we constructed three non-mutually exclusive variables representing report of (1) one or more childhood physical assaultive traumas (CPAT; stabbed, shot, mugged, threatened with a weapon, robbed, kidnapped, held captive), (2) childhood sexual assaultive traumas (CSAT; rape or molestation), and (3) childhood non-assaultive traumas (CNAT; life-threatening accident, disaster, witnessing someone seriously injured or killed, unexpectedly finding a dead body), experienced prior to age 13, to ensure that traumatic exposure occurred prior to longitudinal EEGc measures. CPAT, CSAT, and CNAT were endorsed by 8.65%, 43.93%, and 25.24% of the sample, respectively. Based on literature that suggests socioeconomic status (SES) can impact brain development (Hackman, Farah, & Meaney, 2010), we covaried for family SES as indexed by parental report of highest education in COGA Phase 1–3.

*EEG Recording and Data Processing* procedures have been detailed previously (Meyers et al., 2023) and are summarized in the Supplementary materials.

### Statistical analyses

**Figure 2.**
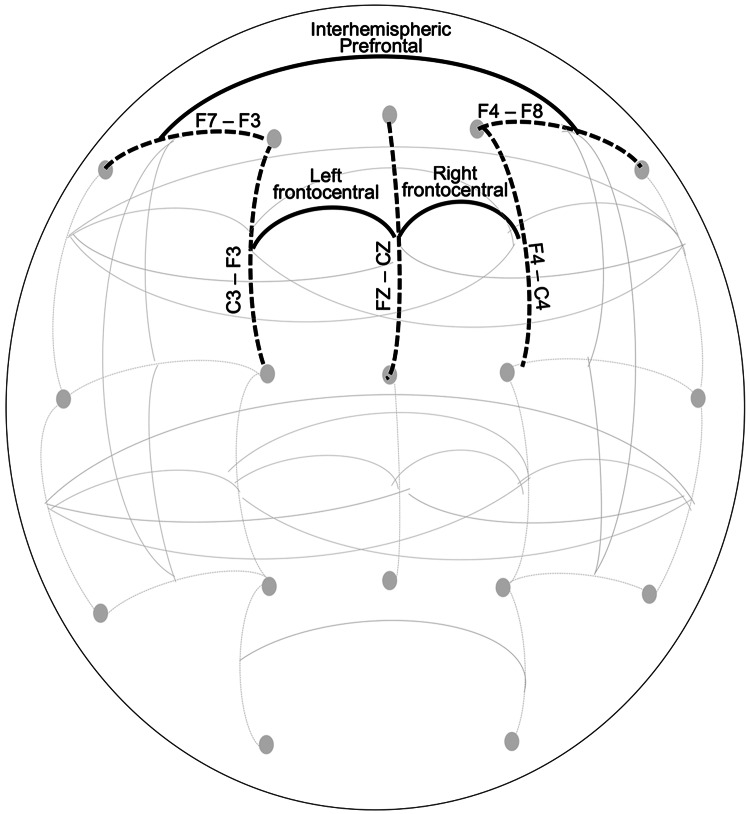
This figure displays a schematic of bipolar electrode pairs (indicated by black dotted lines) and coherence pairs (indicated by black solid lines) derived between bipolar electrode pairs.

All analyses were stratified by sex and clustered by family to account for relatedness. Primary analyses examined discrete childhood trauma variables (CPAT, CSAT, CNAT) as predictors of intercept and slope of EEGc (alpha EEG inter- and intra-hemispheric coherence) during adolescence and young adulthood using latent growth curve models (LGCM) for those with three or more EEG assessments. We ran the LGCM models in Mplus version 8.9 (Muthén & Muthén, 1998-2017), which employs full information maximum likelihood to account for missing data. A non-response analysis indicated that individuals who did not return for follow-up were younger (OR: 2.1, *p* < 0.001) and less likely to report a prior non-assaultive trauma (OR: 1.3, *p* < 0.001). No significant differences regarding sex, race/ethnicity, sexual and assaultive trauma exposure, or EEGc were observed. EEGc from bipolar pairs at three frontal sites (Fig. 2), left intra-hemispheric frontal-central (LFC: FZ-CZ--F3-C3), right intra-hemispheric frontal-central (RFC: FZ-CZ--F4-C4), and prefrontal inter-hemispheric (PFI: F8-F4--F7-F3) were examined in separate models. We modeled both linear and quadratic growth components for EEGc. The models that included the quadratic term did not converge, thus we moved ahead with a linear slope term. Any mention of slope in the following sections refer to linear slope.

**Figure 3.**
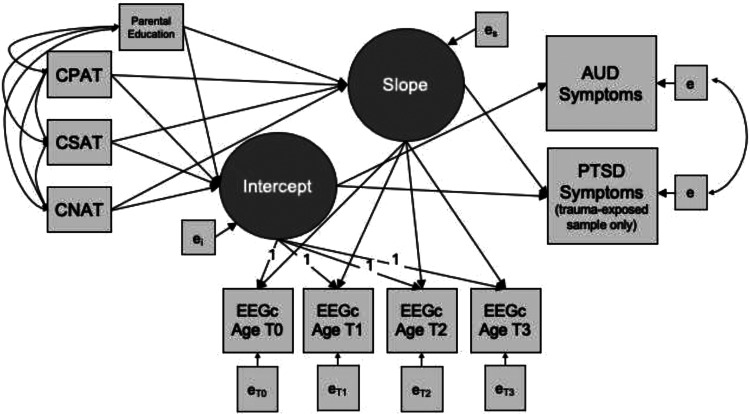
This figure displays a path diagram of the latent growth curve model used to evaluate the association between childhood trauma and intercept and slope of EEG coherence, as well as associations between EEG coherence intercept and slope with AUD symptoms. In the trauma-exposed subsample, PTSD symptoms were also examined as a dependent variable, with the correlation between AUD and PTSD symptoms accounted for by the model. Growth curves were estimated based on individually varying age at the time of each EEG coherence observation. Parental education was included as a covariate. *Note*: CNAT, Childhood non-assaultive trauma; CSAT, Childhood sexual assaultive trauma; CPAT, Childhood physical assaultive trauma; EEGc, EEG coherence.

Next, we examined the association between childhood trauma exposure and the intercepts and slopes of EEGc. The intercept estimates individual variation in starting values, which may reflect different types of exposures prior to the measurement period, as well as other trait differences. The slope reflects how change in EEGc occurs over time, on average. Given the known co-occurrence of different types of trauma exposures, the models allowed for simultaneous testing of associations between trauma exposure types and EEGc intercept and slope. The models also evaluated whether intercept and growth parameters in EEGc were associated with AUDsx in the full sample. In a subset of the sample that endorsed exposure to a potentially traumatic event (i.e. met Criterion A for PTSD diagnosis) at any point from baseline to follow-up 3, we added PTSDsx as an additional dependent variable to account for the co-occurrence of AUD and PTSD. Figure 3 is an illustration of the path diagram for the analytic model.

## Results

### Descriptive characteristics of the sample, stratified by sex

Demographic, alcohol and trauma-related characteristics of the sex-stratified sample are provided in Table 1. The overall sample (*N* = 1652) was 51.75% female. There were no sex differences among race/ethnic groups or participation rates. At baseline, females were significantly younger than males, but mean age did not significantly differ at the three follow-up assessments. Male participants reported significantly more lifetime maximum drinks, and AUD symptoms. Although male and female participants did not differ on rates of childhood trauma exposure, male participants were more likely to report CPAT, whereas female participants were more likely to report CSAT. Among participants exposed to any traumatic event prior to follow-up 3, mean PTSD symptoms 0.75 in males and 1.56 in females, and the modal response for both males and females was zero symptoms. Female participants reported significantly more PTSD symptoms and significantly fewer AUD symptoms compared to male participants.

**Table 1.** Descriptive information for demographic, alcohol-related, and trauma-related characteristics of the sample

Mean (s.d.) or N (%)	Male baseline *N* = 797	Female baseline *N* = 855	*z/t/χ*^2^	*p*
Race/ethnicity				
White	468 (58.72)	506 (63.49)	−0.19	0.849
Black/African American	251 (31.49)	277 (32.40)	−0.39	0.697
Asian	3 (0.38)	4 (0.47)	−0.29	0.771
Hispanic	104 (13.05)	99 (11.58)	0.91	0.363
N at follow-up 1	653 (81.93)	701 (81.99)	−0.03	0.976
N at follow-up 2	461 (57.84)	508 (59.42)	−0.65	0.516
N at follow-up 3	319 (40.02)	366 (42.81)	−1.15	0.250
Age at baseline	14.44 (1.54)	14.16 (1.49)	−3.76	***<0.001***
Age at follow-up 1	17.82 (2.85)	17.74 (2.70)	−0.59	0.558
Age at follow-up 2	20.41 (3.18)	20.51 (3.40)	0.62	0.538
Age at follow-up 3	22.74 (3.31)	22.39 (3.85)	−1.98	0.050
Parental education (years)	13.16 (2.21)	13.04 (2.14)	−1.03	0.304
Ever drink	660 (82.81)	707 (82.69)	0.07	0.952
Regular drinker	542 (68.01)	566 (66.20)	0.78	0.435
Maximum drinks	14.90 (12.71)	9.85 (9.79)	−9.08	***<0.001***
Onset age of regular drinking	17.48 (2.49)	17.85 (2.43)	3.06	**0.002**
AUD symptoms (full sample)	1.45 (2.09)	1.03 (1.87)	−4.31	***<0.001***
Any childhood trauma	258 (32.37)	249 (29.12)	1.43	0.153
Non-assaultive trauma	216 (27.10)	201 (23.51)	1.68	0.093
Physical-assaultive trauma	99 (12.42)	44 (5.15)	5.25	***<0.001***
Sexual-assaultive trauma	11 (1.38)	54 (6.32)	−5.16	***<0.001***
Any trauma by follow-up 3	408 (51.19)	459 (53.68)	−5.06	***<0.001***
PTSD symptoms*	0.75 (2.60)	1.56 (3.84)	3.59	***<0.001***
AUD symptoms*	1.20 (1.91)	0.95 (1.72)	−2.03	**0.043**

*Note:* AUD, alcohol use disorder, and PTSD, post-traumatic stress disorder. PTSD symptoms were assessed only in those who reported a history of trauma. Maximum drinks, AUD symptoms, PTSD symptoms were lifetime measures assessed at follow-up 3. *These items (PTSD and AUD symptoms) were calculated among individuals who reported any trauma exposure by follow-up 3. **Bold** text indicates *p*-value less than 0.05. ***Bold italic*** text indicates *p*-value less than 0.01.

### EEG functional connectivity patterns and associations with childhood trauma

Results of LGCMs estimating the unconditional mean intercept and linear slope of EEGc over time are displayed in Table 2. There was significant positive mean intercept and slope of all three frontal alpha EEGc pairs, indicating that overall, coherence tended to increase over time. The association of CPAT, CSAT, and CNAT with intercept and slope of frontal alpha EEGc, and the association between slope and intercept with AUD symptoms in male and female participants are displayed in Table 3. In the male-only model, we observed a significant association between CPAT and intercept (ß = −0.17, *p* = 0.010) and slope (ß = 0.11, *p* = 0.006) of LFC alpha EEGc (FZ-CZ--F3-C3). This suggests that males who reported CPAT had lower values at baseline, but a faster rate of change in EEGc over time. This association was not observed in male RFC or PFI alpha EEGc (FZ-CZ--F4-C4 and F8-F4--F7-F3, respectively). In contrast, in female participants, we observed a significant association between CSAT and intercept (ß = 0.13, *p* = 0.008) and slope (ß = −0.07, *p* = 0.016) of LFC alpha EEGc (FZ-CZ--F3-C3), as well as the intercept (ß = 0.16, *p* = 0.002) and slope (ß = −0.07, *p* = 0.024) of PFI alpha EEGc (F8-F4--F7-F3). This indicates that female participants who reported CSAT had higher initial values of LFC and PFI alpha EEGc, and a decreased rate of change in LFC and PFI alpha EEGc over time. We observed no significant associations between CNAT and EEGc, and no associations between CSAT or CPAT on our measure of RFC alpha EEGc (FZ-CZ--F4-C4). Greater parental education was associated with decreased intercept (ß = −0.02, *p* = 0.003) of LFC alpha EEGc in males but had no association with intercept or slope in females.

**Table 2.** Results of the unconditional linear growth model for repeated measures of three frontal alpha EEG coherence Pairs in adolescent male and female COGA participants

	Left frontal-central (LFC) EEG coherence FZ-CZ--F3-C3				Right frontal-central (RFC) EEG coherence FZ-CZ--F4-C4				Prefrontal interhemispheric (PFI) EEG coherence F8-F4--F7-F3			
	Males		Females		Males		Females		Males		Females	
	Beta (s.e.)	*p*	Beta (s.e.)	*p*	Beta (s.e.)	*p*	Beta (s.e.)	*p*	Beta (s.e.)	*p*	Beta (s.e.)	*p*
**Unconditional linear growth model**												
Intercept Mean	0.41 (0.02)	***<0.001***	0.46 (0.02)	***<0.001***	0.43 (0.02)	***<0.001***	0.46 (0.02)	***<0.001***	0.22 (0.02)	***<0.001***	0.26 (0.02)	***<0.001***
Slope mean	0.01 (0.00)	***<0.001***	0.01 (0.00)	***<0.001***	0.01 (0.00)	***<0.001***	0.01 (0.00)	***<0.001***	0.00 (0.00)	***0.001***	0.00 (0.00)	***0.003***
Intercept Variance	0.04 (0.01)	***0.005***	0.01 (0.00)	***0.001***	0.02 (0.01)	**0.131**	0.03 (0.01)	***0.001***	0.01 (0.01)	**0.024**	0.03 (0.00)	***<0.001***
slope Variance	0.00 (0.00)	***0.004***	0.00 (0.00)	***<0.001***	0.00 (0.00)	***0.009***	0.00 (0.00)	***<0.001***	0.00 (0.00)	***<0.001***	0.00 (0.00)	***<0.001***
Intercept-slope Correlation	−0.00 (0.00)	**0.012**	0.00 (0.00)	***0.001***	−0.00 (0.00)	0.132	−0.00 (0.00)	***0.001***	0.00 (0.00)	**0.028**	−0.00 (0.00)	***<0.001***
Observations	797	–	855	–	797	–	855	–	797	–	855	–
AIC	−1872.95	–	1268.54	–	−1861.41	–	−1259.75	–	−2135.71	–	−1876.17	–
BIC	−1830.82	–	−1225.78	–	−1819.28	–	−1216.99	–	−2093.58	–	−1833.41	–
H_0_ log-likelihood	945.48	–	643.27	–	939.70	–	638.88	–	1076.86	–	947.09	–
H_1_ maximum log-likelihood	952.62	–	642.37	–	954.66	–	637.40	–	1091.47	–	949.60	–

Note: **Bold** text indicates *p*-value less than 0.05. ***Bold italic*** text indicates *p*-value less than 0.01.

**Table 3.** Linear growth model results for the effect of childhood trauma exposure on slope and intercept of three frontal alpha EEG coherence Pairs and subsequent AUD symptoms in adolescent male and female COGA participants

	Left frontal-central (LFC) EEG coherence FZ-CZ--F3-C3				Right frontal-central (RFC) EEG coherence FZ-CZ--F4-C4				Prefrontal interhemispheric (PFI) EEG coherence F8-F4--F7-F3			
	Males		Females		Males		Females		Males		Females	
	Beta (s.e.)	*p*	Beta (s.e.)	*p*	Beta (s.e.)	*p*	Beta (s.e.)	*p*	Beta (s.e.)	*p*	Beta (s.e.)	*p*
**Associations with intercept and slope of alpha EEG coherence**												
CNAT on intercept	0.00 (0.05)	0.960	−0.04 (0.03)	0.240	0.01 (0.04)	0.727	−0.02 (0.02)	0.382	−0.03 (0.02)	0.058	−0.02 (0.03)	0.552
CSAT on intercept	0.16 (0.12)	0.184	0.13 (0.05)	**0.008**	0.21 (0.13)	0.094	0.07 (0.12)	0.558	−0.01 (0.05)	0.830	0.16 (0.05)	**0.002**
CPAT on intercept	−0.17 (0.07)	**0.010**	−0.06 (0.08)	0.494	−0.05 (0.06)	0.381	−0.01 (0.06)	0.857	−0.00 (0.02)	0.972	−0.05 (0.08)	0.52
Parental education on intercept	−0.02 (0.01)	**0.003**	−0.02 (0.01)	0.069	−0.01 (0.01)	0.078	−0.01 (0.06)	0.149	−0.00 (0.00)	0.575	−0.01 (0.01)	0.079
CNAT on slope	0.00 (0.03)	0.886	0.01 (0.02)	0.771	−0.01 (0.02)	0.820	0.00 (0.01)	0.908	0.02 (0.01)	0.102	−0.00 (0.02)	0.937
CSAT on slope	−0.05 (0.06)	0.394	−0.07 (0.03)	**0.016**	−0.09 (0.07)	0.183	−0.02 (0.06)	0.719	0.05 (0.03)	0.064	−0.07 (0.03)	**0.024**
CPAT on slope	0.11 (0.04)	**0.006**	0.04 (0.05)	0.450	0.04 (0.03)	0.236	0.00 (0.03)	0.933	−0.01 (0.01)	0.592	0.05 (0.05)	0.310
Parental education on slope	0.01 (0.01)	0.057	0.01 (0.01)	0.449	0.00 (0.00)	0.402	0.00 (0.00)	0.897	−0.00 (0.00)	0.422	0.00 (0.00)	0.910
**Associations with AUD symptoms**												
Intercept on AUD symptoms	−0.14 (0.43)	0.745	−0.21 (0.70)	0.770	0.36 (0.18)	**0.039**	0.23 (1.79)	0.897	−3.33 (0.05)	***<0.001***	0.26 (0.47)	0.589
Slope on AUD symptoms	−0.60 (0.51)	0.238	−1.81 (0.73)	**0.013**	−1.35 (1.12)	0.229	−5.94 (13.33)	0.656	1.89 (1.61)	0.42	−1.13 (1.06)	0.288
Observations	797	–	855	–	797	–	855	–	797	–	855	
AIC	4729.54	–	5802.46	–	4748.93	–	5829.92	–	4480.00	–	5196.12	
BIC	4898.05	–	5973.50	–	4917.44	–	6000.96	–	4648.51	–	5367.16	
H_0_ log-likelihood	−2328.77	–	−2865.23		−2338.46	–	−2878.96		−2204.00	–	−2562.06	
H_1_ maximum log-likelihood	−1819.08	–	−2310.33		−1829.38	–	−2321.86		−1696.35	–	−2004.76	

*Note*: CNAT, Childhood non-assaultive trauma; CSAT, Childhood sexual assaultive trauma; CPAT, Childhood physical assaultive trauma. **Bold** text indicates *p*-value less than 0.05. ***Bold italic*** text indicates *p*-value less than 0.01.

### Associations between EEG functional connectivity and AUD symptoms

Results of LGCMs estimating the association between intercept and slope of frontal alpha EEGc at three frontal bipolar pairs with AUDsx in male and female participants are displayed in the lower half of Table 3. In males, we observed a significant positive association between intercept and AUDsx in RFC alpha EEGc (ß = 0.36, *p* = 0.04), and a negative association between intercept and AUDsx in PFI alpha EEGc (ß = −3.33, *p* < 0.001). EEGc These results suggest that both higher initial values in LFC alpha EEGc, but greater initial values PFI alpha EEGc were associated with increased AUDsx. In female participants, we observed a significant negative association with slope of LFC alpha EEGc (ß = −1.81, *p* = 0.013), which suggests steeper decline in LFC alpha EEGc over time was associated with increased AUDsx. We observed no associations between intercept and EEGc on AUDsx in females, and no association between slope and AUDsx in males.

### Association between EEG functional connectivity and AUD and PTSD symptoms in trauma-exposed participants

Results of LGCMs estimating the associations between intercept and slope of alpha EEGc and subsequent AUDsx and PTSDsx in male and female trauma-exposed participants are displayed in Table 4. We found that intercept of LFC alpha EEGc (FZ-CZ--F3-C3) was positively associated with both AUDsx (ß = 0.53, *p* < 0.001) and PTSDsx (ß = 2.65, *p* < 0.001) in trauma-exposed females, suggesting that greater initial values of EEGc were associated with higher levels of PTSD and AUD symptoms. There were no FZ-CZ--F3-C3 intercept or slope associations with AUDsx or PTSDsx observed in trauma-exposed males. For our measure of RFC alpha EEGc (FZ-CZ--F4-C4), we found that intercept was significantly positively associated with both AUDsx (ß = 4.10, *p* < 0.001) and PTSDsx (ß = 2.47, *p* < 0.001) in males. In females, slope of RFC alpha EEGc (FZ-CZ--F4-C4) was negatively associated with AUDsx (ß = −3.16, *p* = 0.002) and PTSDsx (ß = −5.19, *p* = 0.001), suggesting that diminished growth of EEGc was associated with greater AUDsx and PTSDsx. For PFI alpha EEGc (F8-F4--F7-F3), intercept was significantly positively associated with PTSD symptoms in males (ß = 1.05, *p* < 0.001), and females (ß = 1.69, *p* < 0.001). Slope of the same coherence pair (F8-F4--F7-F3), was negatively associated with PTSDsx (ß = −2.02, *p* = 0.0231) in trauma-exposed males, suggesting that higher initial values and diminished growth of PFI alpha EEGc associated with increased PTSDsx.

**Table 4.** Linear growth model results measuring associations between slope and intercept of three frontal alpha EEG coherence pairs and subsequent AUD symptoms in the trauma-exposed subsample of adolescent male and female COGA participants

	Left frontal-central (LFC) EEG coherence FZ-CZ--F3-C3				Right frontal-central (RFC) EEG coherence FZ-CZ--F4-C4				Prefrontal interhemispheric (PFI) EEG coherence F8-F4--F7-F3			
	Males		Females		Males		Females		Males		Females	
	Beta (s.e.)	*p*	Beta (s.e.)	*p*	Beta (s.e.)	*p*	Beta (s.e.)	*p*	Beta (s.e.)	*p*	Beta (s.e.)	*p*
**Associations with AUD and PTSD symptoms**												
Intercept on AUD symptoms	0.08 (0.45)	0.862	0.53 (0.12)	***<0.001***	4.10 (0.19)	***<0.001***	−0.58 (0.66)	0.377	0.41 (0.60)	0.494	0.53 (1.10)	0.633
Slope on AUD symptoms	−0.91 (0.77)	0.237	−0.79 (0.77)	0.305	−1.55 (1.26)	0.218	−3.16 (1.03)	***0.002***	−1.39 (0.83)	0.094	−0.22 (0.58)	0.700
Intercept on PTSD symptoms	0.28 (0.15)	0.066	2.65 (0.12)	***<0.001***	2.47 (0.09)	***<0.001***	0.15 (0.18)	0.419	1.05 (0.13)	***<0.001***	1.69 (0.08)	***<0.001***
Slope on PTSD symptoms	0.01 (0.48)	0.992	−0.48 (0.94)	0.612	−0.22 (0.56)	0.699	−5.19 (1.52)	***0.001***	−2.02 (0.89)	**0.023**	−0.82 (0.55)	0.137
Correlation between AUD and PTSD symptoms	0.01 (0.01)	0.312	0.01 (0.01)	0.552	0.00 (0.01)	0.863	0.01 (0.01)	0.494	−0.00 (0.01)	0.887	0.00 (0.01)	0.718
Observations	408	–	459	–	408	–	459	–	408	–	459	–
AIC	3034.34	–	3950.75	–	3061.07	–	3934.99	–	2900.58	–	3497.02	–
BIC	3198.81	–	4120.04	–	3225.54	–	4104.28	–	3065.04	–	3666.31	–
H_0_ log-likelihood	−1476.17	–	−1934.37	–	−1489.537	–	−1926.49		−1409.29	–	−1707.51	–
H_1_ maximum log-likelihood	−1037.24	–	−1495.84	–	−1057.53	–	−1489.05		−970.62	–	−1261.84	–

*Note*: Analyses were limited to individuals who reported at least one traumatic experience during any assessment from baseline to the third follow-up. PTSD symptoms and AUD symptoms represent the lifetime maximum symptoms as reported at the third follow-up assessment. Models account for childhood trauma variables and parental education associations with intercept and slope of EEG coherence. **Bold** text indicates *p*-value less than 0.05. ***Bold italic*** text indicates *p*-value less than 0.01.

## Discussion

This is the first study, to our knowledge, to examine the association between childhood trauma and longitudinal EEG coherence (EEGc) across adolescence and its role as a potential biological mechanism linking trauma exposure to AUD and PTSD symptoms in young adulthood. We observed differences in trajectories of left frontocentral alpha EEGc related to childhood sexual and physical assaultive trauma in females and childhood physical assaultive trauma in males. The left frontotemporal region of the brain is associated with language, learning, and memory, and has been implicated in studies of the effect of traumatic stress on the brain (Bremner, 2006; Carrion, Weems, Richert, Hoffman, & Reiss, 2010; Carrion & Wong, 2012; Stark et al., 2015). EEG interhemispheric alpha coherence in the prefrontal region also showed increased intercept but diminished growth in females with history of childhood sexual assaultive trauma, suggesting differences in executive function in these individuals. EEGc was associated with subsequent AUD and PTSD symptoms in trauma-exposed participants, with patterns indicating that trauma exposure was associated with worse AUD/PTSD outcomes.

As hypothesized, we found different patterns of EEGc over time related to childhood trauma exposure before age 13. Results demonstrating differences in both intercept and slope of EEGc are associated with childhood assaultive trauma and psychopathology extend the largely cross-sectional, adult-focused literature (Cardenas, Price, & Fein, 2018; Cook et al., 2009; Park et al., 2017; Tcheslavski & Gonen, 2012). Our models indicated higher baseline left frontocentral EEGc in females with CSAT, consistent with a small (*n* = 30) cross-sectional study by Ito, Teicher, Glod, and Ackerman (1998), which observed higher alpha EEGc in the left hemisphere of children with severe sexual or physical abuse histories. The present study extends this literature by demonstrating nuanced trajectories: decreased alpha EEGc growth in females with CSAT and increased growth in males with CPAT. One explanation for these findings is that childhood trauma may lead to extended hyperarousal and mesolimbic system overactivation, disrupting typical neural network development (Teicher et al., 1997; Teicher & Samson, 2016). Notably, no associations between CNAT and EEGc were observed, consistent with literature indicating assaultive trauma poses higher risk for PTSD (Breslau et al., 1998), depression (McCutcheon et al., 2009), and more pronounced changes in brain development (De Bellis & Zisk, 2014; Meyers et al., 2019b). Additionally, this study extends literature on functional connectivity and shared liability for AUD and PTSD. Differences in frontal functional connectivity were associated with AUD and PTSD symptoms, but to varying degrees across sexes. Importantly, findings demonstrated associations with both slope and intercept, offering insights potentially missed in prior cross-sectional studies. Together, these findings on trauma, AUD, and EEGc offer some of the first longitudinal evidence of associations between childhood trauma and frontal neural connectivity, with implications for understanding development of psychopathology.

The present study observed significant associations between childhood trauma and alpha EEGc in only the left hemisphere for both males and females, regardless of trauma type. Previous studies have reported differences between hemispheres in individuals with childhood trauma, such as smaller left dorsolateral prefrontal cortex volume in those with childhood trauma (Carballedo et al., 2012; Lu et al., 2019; Paquola, Bennett, & Lagopoulos, 2016). While our study investigated connectivity in the frontal and central regions of the brain, other studies have seen lateral differences in the temporal region, such as smaller left hippocampal volume compared to controls (Bremner et al., 1997; Shu et al., 2013; Stein, Koverola, Hanna, Torchia, & McCLARTY, 1997). Further, less hippocampal activation is associated with increased PTSD symptoms (van Rooij et al., 2018). Another study observed abnormal connectivity between the left PFC and anterior hippocampus in children with PTSD (Heyn et al., 2019). Given the findings from the present study and previous connectivity and imaging studies, future studies should investigate the associations between sex differences and trauma type with connectivity between the PFC and hippocampus in individuals reporting childhood trauma.

Sex differences in trauma type, PTSD, and AUD, are well documented by previous research (Beals et al., 2013; Boudoukha, Ouagazzal, & Goutaudier, 2017; Chung & Breslau, 2008; Erol & Karpyak, 2015; Kessler, Sonnega, Bromet, Hughes, & Nelson, 1995). Men are more likely to experience trauma, but less likely to be diagnosed with PTSD compared to women (Beals et al., 2013; Kessler et al., 1995). Women are more likely to have co-morbid PTSD and AUD (Peltier et al., 2022), potentially due to higher risk of recurring, high impact trauma (Beals et al., 2013; Hien, Cohen, & Campbell, 2005). In our sample, CSAT was more prevalent in females, and CPAT more prevalent in males. Females also reported significantly higher PTSD symptoms, which may be related to their higher rates of CSAT. Moreover, sex may play also influence the relationship between trauma and neurocognitive development (Helpman et al., 2017). Prior research in COGA (Meyers et al., 2019b) found that females reporting sexual assault had a decreased rate of change in frontal theta oscillations during response inhibition. Structural and functional neuroimaging studies also demonstrate interactions among sex, type, and timing of trauma exposure (for review, see Helpman et al., 2017). In the present study, we observed sex differences dependent on trauma type: in males, CPAT was linked to lower baseline left frontocentral alpha EEGc and a faster rate of change, while in females, CSAT was linked to higher starting values and a slower rate of change. While the observed sex differences may relate to the variation in physical v. assaultive trauma prevalence, the differences in direction of these associations may also suggest true sex-specific effects.

The findings from this study have important implications. First, these results suggest that traumatic experiences in childhood have lasting impacts on brain function, as evidenced by the fact that sexual/physical abuse was associated with both concurrent influences on EEGc and on changes across development. Additionally, type of trauma experienced may have differential associations with EEGc across sexes. Females with childhood sexual assault exposure and males with childhood physical assault exposure may be particularly high-risk groups on whom prevention and intervention efforts should be prioritized. Although links between trauma and PTSD and AUD have been well-established, these study results begin to suggest that atypical changes in alpha EEGc across development may be one factor by which certain traumatic experiences confer risk for AUD and PTSD. This study joins other emerging work (e.g. Kummar, Correia, and Fujiyama, 2019) in suggesting that there may be some clinical value in integrating EEG measures into treatment for trauma-exposed individuals, as such biological markers may enable earlier diagnoses and/or an avenue through which to provide neurofeedback over the course of treatment.

### Limitations and future directions

#### Limitations

Missing data – LGCM requires at least three timepoints, which were present in ~85% of COGA's prospective study sample overall, but only 59% of the analytic sample in the present analyses. Childhood Trauma Measures – While some traumatic events would have occurred during the study period (baseline through follow-up 3), many of the childhood traumatic events assessed relied on retrospective self-reports. Retrospective reports may be unstable over time, typically underestimating trauma exposure prevalence, potentially biasing this study's findings towards the null, especially for the individuals who were older at baseline. This may also account in part for the relatively small effect sizes observed for the effect of trauma on EEGc. Additionally, participants’ perceptions of how stressful they found the traumatic event, experiences of prolonged/repeated traumas, and childhood maltreatment (e.g. neglect) were not measured. Although our models accounted for the effect of different types of trauma, we did not present the effect of multiple types of trauma exposure (e.g. CPAT*CSAT) due to power limitations, despite evidence that children often experience multiple types of childhood abuse (Chiu et al., 2013). Future analyses that account for ‘dose’ effects (e.g. an individual who has been stabbed, shot, and mugged would have a CPAT dose of 3) and interactive effects would add further insight into our understanding of the effects of complex trauma on the developing brain. Additionally, as symptom counts reflected lifetime problems, it is unknown whether the symptoms reported reflect participants’ current state or whether they had recovered by the time of assessment. Confounding variables – This was an observational study, so we caution against any causal inferences based on the significant associations in our findings, as these associations could be attributed to third variables. There are several factors unaccounted for in the present analyses that may impact the neurodevelopmental processes examined, such as parental factors, sociodemographic factors, fetal alcohol exposure, and individual factors, including genetics and family history of alcohol problems (Pandey et al., 2020). Additional research that more comprehensively integrates the range of important factors is needed.

#### Future directions

This study focused on alpha EEGc, which is just one of several potentially informative EEG phenotypes. The number of EEG phenotypes available is so vast that future research would likely benefit from integrating machine learning and other methods for the management of big data (Golmohammadi, Harati Nejad Torbati, Lopez de Diego, Obeid, & Picone, 2019; Pawan & Dhiman, 2023) while maintaining a focus on clinical relevance when executing these techniques. In addition, studies that more comprehensively assess childhood trauma (e.g. repeated/prolonged trauma), maltreatment (e.g. neglect), and other stressful life experiences (e.g. discrimination, neighborhood violence) are needed to better understand the way that adverse experiences in childhood may impact neural connectivity and subsequent psychopathology. Another important future direction for this research is the integration of genetic and other biological factors (e.g. family history) associated with trauma, PTSD, and alcohol-related outcomes (e.g. consumption, problems, AUD) and EEG connectivity. Including these informative measures may shed further light on the shared neurobiological mechanisms of risk between PTSD and AUD, as well as increase our ability to better identify individuals who may benefit most from intervention.

## Conclusions

This novel study explored associations between childhood trauma and longitudinal alpha EEG coherence in frontal brain regions in children and adolescents, and their association with subsequent AUD and PTSD in young adulthood. Our findings suggest that childhood assaultive trauma is associated with changes in neural connectivity patterns, and the nature of these associations differs by sex and trauma exposure type. Further, trajectories of neural connectivity were associated with subsequent AUD and PTSD symptoms. These findings contribute valuable insights into the neurodevelopmental consequences of childhood trauma, extending beyond the predominantly cross-sectional adult literature. Importantly, sex-specific variations in neural connectivity underscore the need for a comprehensive understanding of trauma's impact on brain functioning. The relevance of EEG coherence as a potential biomarker for early diagnosis and targeted interventions is also emphasized. Moving forward, integrating machine learning techniques and exploring other genetic and biological factors could enhance our understanding of the shared neurobiological mechanisms underlying PTSD, AUD, and trauma-related EEG coherence, with the goal to guide targeted interventions for high-risk groups.

## Supporting information

Neale et al. supplementary material Neale et al. supplementary material

## Supplementary material

The supplementary material for this article can be found at https://doi.org/10.1017/S0033291724002599.
