# Supplementary material for: Childhood trauma is associated with developmental trajectories of EEG coherence, alcohol-related outcomes, and PTSD symptoms
Source: Psychol Med. 2024 Dec 2;54(15):4302–15. doi: 10.1017/S0033291724002599 (PMC11650155; doi:10.1017/S0033291724002599)
Supplement: Neale et al. supplementary material [file S0033291724002599sup001.docx]

**Supplementary Materials**

*EEG Recording and Data Processing.* Briefly, resting (eyes-closed) EEG was recorded for 4.25 min; a continuous interval of 256 seconds was analyzed. Each subject wore a fitted electrode cap containing a 61-channel montage of scalp electrodes (Electro-Cap International Inc). The nose served as reference and the ground electrode was placed on the forehead. Eye movements are monitored with electrodes above and below left eye. EEG was recorded with the subjects seated comfortably in a dimly lit sound-attenuated temperature-regulated booth. They were instructed to keep their eyes closed and remain relaxed, but not to fall asleep. Electrode impedances were maintained below 5 kΩ. EEG activity was amplified by a factor of 10,000 on Neuroscan amplifiers (Synamps2), filtered between 0.02 Hz and 100 Hz and sampled at 500 Hz using the Neuroscan software system running on 186 PCs. Identical procedures were performed at all collection sites at baseline and each follow-up assessment. Digitized electrophysiological data underwent quality control and were edited for known artifacts (movement, EMG, eye movement, DC shifts); filtering and ocular correction were implemented where required. Between 19-64 channels according to the 10-20 International system were used for analysis. Bipolar electrode pairs were derived to reduce volume conduction effects. Conventional Fourier transform methods were used to calculate EEG interhemispheric and intrahemispheric coherence at 27 bipolar pairs in the following frequency bands: theta (3-7 Hz), alpha (8-12 Hz), and beta (13-20 Hz). Additional information on EEG methods are available elsewhere (Meyers, Brislin et al., 2023).
